# Dataset on the optimization by response surface methodology for the synthesis of silver nanoparticles using Laxitextum bicolor mushroom

**DOI:** 10.1016/j.dib.2022.108631

**Published:** 2022-09-23

**Authors:** Krishia Rei A. Javier, Drexel H. Camacho

**Affiliations:** a Department of Chemistry, De La Salle University, 2401 Taft Avenue, Manila 0922, Philippines; b Central Instrumentation Facility, De La Salle University – Laguna Campus, Laguna Boulevard, LTI Spine Road, Barangays Biñan and Malamig, Biñan, Laguna 4024, Philippines

**Keywords:** Central composite design, Laxitextum bicolor, Surface plasmon resonance, Lack of fit, Silver nanoparticle

## Abstract

Silver nanoparticles (AgNP) are important materials in developing novel devices owing to their unique physical and chemical properties. It attracted much interest because it exhibits a prominent Surface Plasmon Resonance (SPR) property that is dependent on its nanodimension and nanostructure. Its green synthesis using biological extracts offers a cheap and benign process to afford AgNPs. However, natural extracts contain thousands of metabolites that affect the formation of the desired AgNP. Other factors such as temperature, pH, time, and volume also influence the formation of the nanometal hence, optimization is always carried out to afford sufficient amounts of the nanometals. To eliminate further trials and errors, this work reports the optimization of AgNP using the aqueous extract from Laxitextum bicolor, a wild type of mushroom from the family of Stereacea. Using central composite design (CCD) under Response Surface Methodology (RSM), five levels from each of the five independent parameters (pH, temperature, time, volume of extract, and volume of AgNO_3_) in a single-block mode afforded 32 experimental runs where SPR at 420 nm of the formed AgNP was measured as the dependent variable. ANOVA evaluation revealed that the p-value of the refined model is significant (*p*-value = 0.00) and the *p*-value of lack-of-fit is insignificant (LoF = 0.223). Model statistics displayed acceptable goodness of fit (R^2^ = 98.54%, adjusted R^2^ = 97.34, predicted R^2^ = 92.50%). The predicted optimal condition to synthesize AgNP from aqueous extract of *L. bicolor* were determined to be pH *=* 10*,* Temperature = 55 °C, Time = 180 min, Vol. Ext = 1.5 mL, and Vol. AgNO_3_ = 20 mL. To check the accuracy and repeatability of predicted optimal synthesis conditions, the UV-Vis analysis was employed. It showed that the peak intensity has a narrow peak with an absorbance of 3.40 at around 420 nm, which was the set criteria for choosing the optimal synthesis condition.

**Specifications Table**

**Table:** 

Subject	Materials Science
Specific subject area	Silver nanoparticles
Type of data	Tables and Figures
How the data were acquired	Optimization: Using Minitab software version 17, CCD has been utilized to obtain the best SPR band in peak intensity at 420 nm and used as the response in the design. The design was done based on five levels for each noted parameter in a single-block mode and obtained 32 analysis runs in a random order to minimize the effect of uncontrolled variables on the quality of the AgNP's SPR. The five parameters set as independent variables were: pH, temperature, time, the volume of extract, and the volume of AgNO_3_ solution that greatly affect the dependent variable which was the SPR at 420nm. UV-Vis: The spectra for the peak intensity of SPR were obtained using Shimadzu UV-Visible spectrophotometer UV-2600. Settings were set to 2 nm slit width, medium scan speed, and wavelength range of 250-750nm. Baseline correction using deionized water was employed before running the samples.
Data format	Raw and analyzed
Description of data collection	Silver nanoparticles were synthesized using the predicted optimal condition attained in central composite design. The study used five parameters in optimization using CCD: pH (5.5–11.5), temperature (10–70 °C), time (0–240 min), volume of extract (0.5–4.5 mL), and volume of AgNO_3_ (15–35 mL). The peak intensity was measured for each analysis run at 420 nm via UV-Vis spectroscopy, after following the given conditions. A total of 32 analysis runs were performed and the results were evaluated using the Minitab software. Verification of predicted optimal conditions was performed using UV-Vis spectroscopy.
Data source location	Central Instrumentation Facility-De La Salle University Laguna, Biñan, Laguna, Philippines
Data accessibility	Camacho, Drexel (2022), “Dataset on the optimization by response surface methodology for the synthesis of silver nanoparticles using Laxitextum bicolor mushroom”, Mendeley Data, V2, doi: 10.17632/k3nsmbcrj2.2 Repository name: Mendeley Data Repository Data identification number (permanent identifier, i.e. DOI number): doi: 10.17632/k3nsmbcrj2.2 Direct link to the dataset: https://data.mendeley.com/datasets/k3nsmbcrj2/2

## Value of the Data

• This work presents empirical and statistical data on the optimal conditions identified in the green synthesis of AgNP using Laxitextum bicolor mushroom. The data given here as well as the experimental parameters that were optimized represent the first report on the study of the wild mushroom as well as the utilization of the mushroom extract for the preparation of AgNP. Since each mushroom have their own unique metabolite composition, the data reported herein is specific on Laxitextum bicolor. A generic conclusion for any type of *L. bicolor* or any mushroom species is premature at this point in the absence of data for other mushroom species or types. The metabolites from the *L. bicolor* mushroom have been incorporated in the nanometals as capping agent, which can provide insight into the potential synergistic effect of the AgNP and the metabolite for various applications.
• The results of the central composite design in the optimization protocol for the synthesis of metabolite-capped AgNP eliminate the need for other researchers to do trial and error experiments. The modeling and analysis can be used by other investigators to test and compare the influence of experimental parameters (pH, temperature, time, volume of extract, and volume of the silver nitrate solution) on the synthesis of AgNP especially on larger scale process. The results herein are helpful to researchers who are looking to improve the stability of the colloidal AgNP aqueous solution and increase the yield of AgNP synthesis, especially in bulk production.
• The data presented herein can be used in the optimization of similar green chemistry processes for both small and large scale investigations. These data can be referenced by academic investigators looking at experimental designs in the green synthesis of AgNP and in understanding the interaction of each parameters and their effects on the surface plasmon resonance (SPR) profile. Industry practitioners can benefit from these data especially on exploring the utilization of the metabolite-capped AgNP for antioxidant and antimicrobial applications as well as in wastewater treatment.

## 1 Data Description

**Fig. 1:**
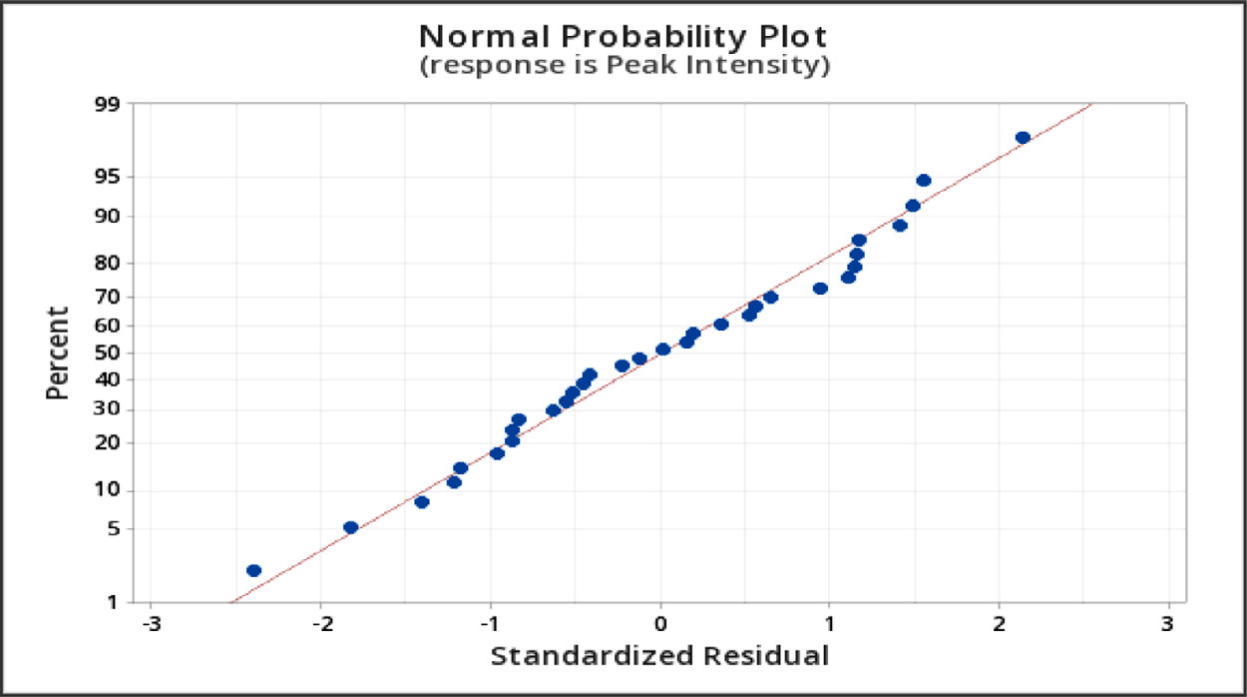
Normal probability plot of standardized residuals for the refined model.

**Fig. 2:**
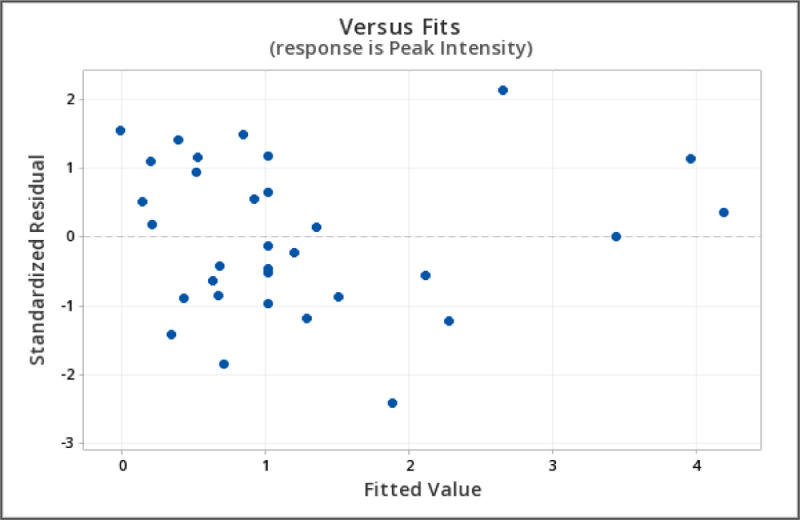
Standard residual vs fitted value of SPR for the refined model.

**Fig. 3:**
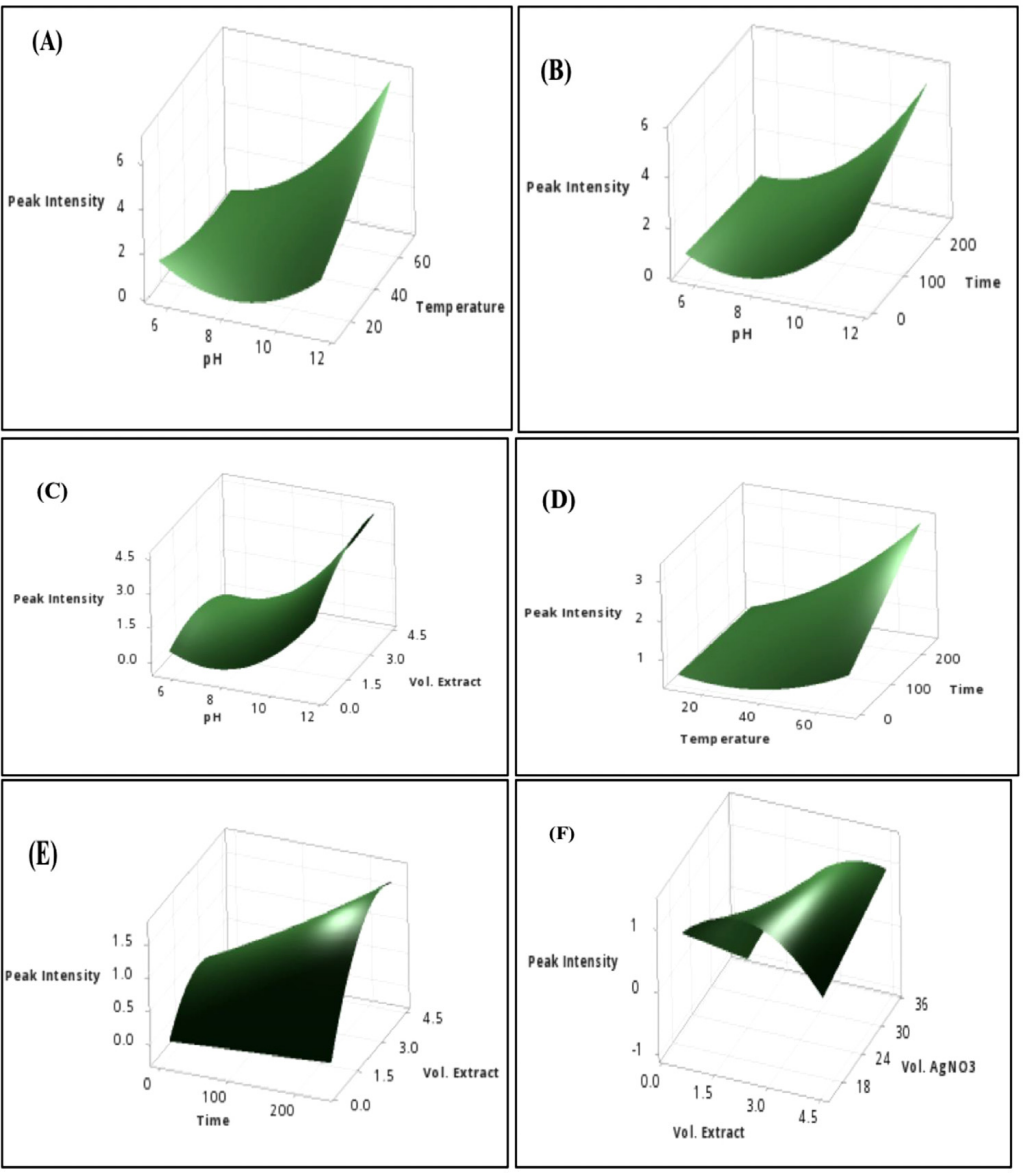
Surface plot of significant model terms.

**Fig. 4:**
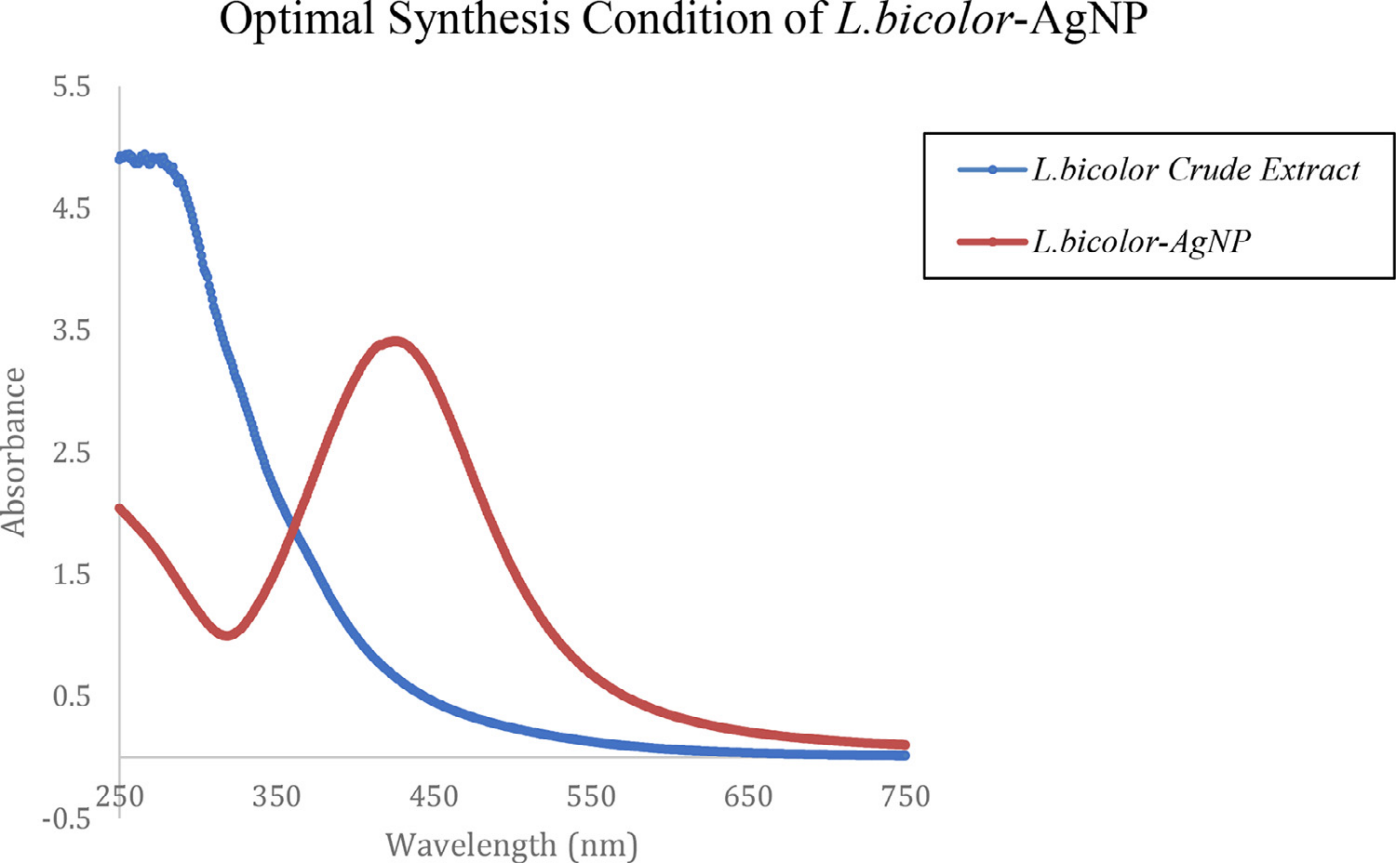
UV-Vis spectra of the optimal condition vs. raw aqueous extract.

The independent variables and the range of variable level for the central composite design are shown in Table 1 where the variables are: pH (5.5–11.5), temperature (10–70 °C), time (0–240 min), volume of extract (0.5–4.5 mL), and volume of AgNO_3_ (15–35 mL). The summary of the 32 runs and the response at 420 nm is shown in Table 2. The raw data can be accessed in Mendeley Data Repository [1]. To determine if the model is a good fit, analysis of variance was utilized. It is also used to identify significant factors that affect the SPR (Table 3). ANOVA revealed that the significant model must have a *p*-value < 0.05 [2,5]. The “Lack of Fit (LOF) F-value and p-value” must be insignificant compared to the pure error, which is a good indication of the model [3,8]. The second-order polynomial equation shown in Eq. (1) describes the process of the full model. In CCD modeling, terms having p-values greater than 0.1 are not significant and must be eliminated from the final selected model at the desired confidence limit [2]. Backward elimination was applied to eliminate the insignificant variables in Eq. (1) and the development of a refined linear model (RM) and Eq. (2) without insignificant variables. Comparison between the FM and RM for the optimization of SPR intensity was done in Table 4. The correlation coefficient of the (R^2^) and adjusted R^2^ (R^2^_adj_) supported the significance of the model and show the goodness of fit in both equations. R^2^ predicted (R^2^_pred_) is used to determine how well a regression model makes a prediction and helps to determine whether the regression model is overfitting. The normal probability plot of standardized residuals and standard residual vs fitted value is shown in Fig. 1, Fig. 2, which are used to confirm the normality of errors-distribution and absence systematic patterns in the values, respectively [2,7]. The surface response plot (Fig. 3) is a graphical representation of regression analysis obtained on the established model. It is used to study the interactions between parameters and their effect on the dependent variable (SPR) and to help in defining the optimum condition of each parameter to give maximum production of AgNP [4]. Response optimization shows the predicted optimal condition for the synthesis of AgNP. The condition was verified using UV-Vis spectroscopy (Fig. 4) and compared to its raw aqueous extract to ensure the repeatability and accuracy of the obtained optimal condition [6]. High intensity with a narrow peak absorbance at 420nm is the criteria set for the best optimal condition.

**Table 1:** Range and Levels of central composite design.

	Variable levels				
Independent Variables	Star low(-2)	Low(-1)	Center (0)	High(+1)	Star High (+2)
pH	5.5	7	8.5	10	11.5
Temperature (˚C)	10	25	40	55	70
Time (min)	0	60	120	180	240
Vol. of Ext (mL)	0.5	1.5	2.5	3.5	4.5
Vol. of 1.0 mM AgNO_3_ (mL)	15	20	25	30	35

**Table 2:** Summary of experiment runs and its peak intensity response at 420 nm absorbance for *L. bicolor* optimization.

Std Order	Run Order	pH	Temperature (˚C)	Time (min)	Vol. Extract (mL)	Vol. AgNO_3_ (mL)	Peak Intensity (absorbance @ 420nm)
23	1	8.5	20	120	0.5	25	0.16569
12	2	10.0	55	60	3.5	20	2.88199
31	3	8.5	40	120	2.5	25	0.85760
13	4	7.0	25	180	3.5	30	0.54736
25	5	8.5	40	120	2.5	15	1.37705
26	6	8.5	40	120	2.5	35	0.61652
8	7	10.0	55	180	1.5	20	3.44737
10	8	10.0	25	60	3.5	30	1.16447
28	9	8.5	40	120	2.5	25	0.99561
27	10	8.5	40	120	2.5	25	1.1245
7	11	7.0	55	180	1.5	30	0.23146
17	12	5.5	40	120	2.5	25	0.56311
1	13	7.0	25	60	1.5	30	0.19605
15	14	7.0	55	180	3.5	20	1.00539
19	15	8.5	10	120	2.5	25	0.63072
11	16	7.0	55	60	3.5	30	0.32032
4	17	10.0	55	60	1.5	30	1.62778
24	18	8.5	40	120	4.5	25	0.50132
32	19	8.5	40	120	2.5	25	0.94108
20	20	8.5	70	120	2.5	25	2.13783
22	21	8.5	40	240	2.5	25	1.3758
16	22	10.0	55	180	3.5	30	4.08624
21	23	8.5	40	0	2.5	25	0.69836
30	24	8.5	40	120	2.5	25	0.93115
14	25	10.0	25	180	3.5	20	2.05509
2	26	10.0	25	60	1.5	20	1.17615
18	27	11.5	40	120	2.5	25	4.23401
5	28	7.0	25	180	1.5	20	0.34080
9	29	7.0	25	60	3.5	20	0.19605
6	30	10.0	25	180	1.5	30	0.98364
3	31	7.0	55	60	1.5	20	0.5773
29	32	8.5	40	120	2.5	25	1.21265

**Table 3:** Analysis of Variance (ANOVA) result of *L. bicolor* optimization for AgNP synthesis.

ANALYSIS OF VARIANCE (ANOVA)										
	DF		Adj. Sum of Square		Adj. Mean of Square		F-Value		P-Value	
Source of Variation	FM	RM	FM	RM	FM	RM	FM	RM	FM	RM
**Model**	20	14	35.47	35.28	1.77	2.52	58.24	82.13	0.00	0.00
**Linear**	5	5	26.54	26.54	5.31	5.31	174.3	173.0	0.00	0.00
pH	1	1	18.99	18.99	18.99	18.99	623.7	619	0.00	0.00
Temperature	1	1	4.62	4.62	4.62	4.62	151.8	150.7	0.00	0.00
Time	1	1	1.46	1.46	1.46	1.46	47.92	47.56	0.00	0.00
Vol. Extract	1	1	0.79	0.79	0.79	0.79	25.87	25.67	0.00	0.00
Vol. AgNO_3_	1	1	0.68	0.68	0.68	0.68	22.38	22.21	0.00	0.00
**Square**	5	3	4.94	4.94	0.99	0.99	32.44	53.63	0.00	0.00
pH*pH	1	1	3.60	3.60	3.60	3.60	118.1	118.2	0.00	0.00
Temp*Temp	1	1	0.27	0.27	0.27	0.27	8.98	8.89	0.012	0.01
Time*Time	1		0.003		0.003		0.1		0.76	
Vol. Ext*Vol. Ext	1	1	0.81	0.81	0.81	0.81	26.59	26.97	0.00	0.00
Vol. AgNO_3_*Vol. AgNO_3_	1		0.00		0.00		0.00		0.99	
**2-Way Interaction**	10	6	3.99	3.99	0.40	0.63	13.09	20.65	0	0.00
pH*Temperature	1	1	2.11	2.11	2.11	2.11	69.28	68.76	0	0
pH*Time	1	1	0.52	0.52	0.52	0.52	17.1	16.97	0.002	0.00
pH*Vol. Extract	1	1	0.31	0.31	0.31	0.31	10.2	10.12	0.009	0.01
pH*Vol. AgNO_3_	1		0.048		0.05		1.57		0.24	
Temp*Time	1	1	0.29	0.29	0.29	0.29	9.66	9.58	0.01	0.01
Temp*Vol. Extract	1		0.08		0.08		2.68		0.13	
Temp*Vol.AgNO_3_	1		0.037		0.037		1.22		0.294	
Time*Vol. Extract	1	1	0.18	0.18	0.18	0.18	5.97	5.92	0.03	0.03
Time*Vol.AgNO_3_	1		0.02		0.0171		0.56		0.47	
Vol. Extract*Vol. AgNO_3_	1	1	0.39	0.39	0.39	0.39	12.65	12.55	0.004	0.002
**Error**	11	17	0.33	0.52	0.03	0.03				
**Lack-of-Fit**	6	12	0.25	0.44	0.041	0.036	2.32	2.04	0.187	0.223
**Pure Error**	5	5	0.09	0.09	0.018	0.018				
**Total**	31	31	35.80	35.80

**Table 4:** Regression of FM and RM.

Model Statistics					
R^2^ Value		R^2^_(Adjusted)_		R^2^_(Predicted)_	
FM	RM	FM	RM	FM	RM
99.06%	98.54%	97.36%	97.34%	81.21%	92.50%

**Eq. (1):** Coded equation for full quadratic model (FM).

**Peak Intensity** = 1.0073+0.8896 [pH]+0.4389 [Temperature] +0.2466 [Time]+0.1812 [Vol. Extract]-0.1685[Vol.AgNO3]+ 0.3501[pH*pH]+ 0.0966[Temperature*Temperature] +0.0101[Time*Time] -0.1661[Vol. Extract*Vol. Extract] -0.0003[Vol. AgNO3*Vol. AgNO3]+ 0.3631[pH*Temperature] +0.1804[pH*Time]+ 0.1393[pH*Vol. Extract] -0.0546[pH*Vol. AgNO3] + 0.1356[Temperature*Time]+0.0715[Temperature*Vol. Extract] -0.0481[Temperature*Vol. AgNO3] +0.1066[Time*Vol. Extract]+ 0.0327[Time*Vol. AgNO3]+ 0.1552 [Vol. Extract*Vol. AgNO3]

**Eq. (2):** Coded equation for refined quadratic model (RM).

**Peak Intensity**= 1.0164+0.8896[pH]+ 0.4389[Temperature]+ 0.2466[Time]+ 0.1812[Vol. Extract] -0.1685[Vol. AgNO_3_] + 0.3494[pH*pH] + 0.0958[Temperature*Temperature] -0.1669[Vol. Extract*Vol. Extract] + 0.3631[pH*Temperature] + 0.1804[pH*Time] + 0.1393[pH*Vol. Extract] +0.1356[Temperature*Time]+ 0.1066[Time*Vol. Extract]+0.1552[Vol. Extract*Vol. AgNO_3_]

## 2 Experimental Design, Materials and Methods

### 2.1 Materials

The materials used for the study are Laxitextum bicolor*,* a wild mushroom collected from Mt. Apo in North Cotabato, Mindanao, silver nitrate (Sigma Aldrich).

### 2.2 Preparation for the Optimization of Conditions Using *L. Bicolor* Extract

The collected hardy mushroom was air-dried, soaked in liquid nitrogen, and crushed using an Osterizer blender. Powdered *L. bicolor* (5.0 g) was soaked in deionized water (150 mL) for 24 h, with continuous stirring at room temperature. The extract was then filtered using Whatman Grade 5 filter paper to separate the solid powder affording a clear light yellow solution.

### 2.3 Synthesis and Purification of Silver Nanoparticles (AgNP)

Silver nitrate solution (1.0 mM) was added in the aqueous extract of *L. bicolor,* which turned the solution from light yellow to dark brown suggestive of the formation of AgNPs. Color change was not observed in the extract solution in the absence of AgNO_3_ over time. To optimize the process of using *L. bicolor* as a green process in the synthesis for AgNP, central composite design (CCD) under response surface methodology (RSM) was conducted, which is reported herein. The following parameters: pH, temperature, time, volume of extract, and volume of 1.0 mM AgNO_3_ were investigated.

The obtained AgNP solution was purified by separating the nanoparticles using DLAB D2012 Plus Centrifuge spinned at 12,000 rpm for 15 min. The supernatant that contains the excess silver ion, nitrate ions, and the extract were discarded. The precipitate, which is the AgNPs, was redispersed in deionized water and was subjected to further centrifugation. The process was done thrice, to ensure that the AgNPs were completely free from any organic molecules from the mushroom extract. The collected AgNPs were dried by lyophilization.

### 2.4 Determination of Surface Plasmon Resonance (SPR) Peak Intensity

The SPR peak intensity was determined spectrophotometrically using Shimadzu UV-Visible spectrophotometer UV-2600 at 420 nm. AgNPs absorb and scatter light with extraordinary efficiency centered at 420 nm. The strong interaction with light at 420 nm occurs because the conduction electrons on the silver nanometal surface undergo a collective oscillation when they are excited by light at this wavelength. A different set of conditions from CCD was performed randomly and the SPR was measured at room temperature.

### 2.5 Experimental Design

#### 2.5.1 Central Composite Design Experiments

The software used in generating the design of experiment and in analyzing the data is Minitab 17 software with institutional license under De La Salle University. central composite design was used, which generated 32 experimental runs. The parameters that served as independent variables were pH (5.5, 7.0, 8.5, 10.0, 11.5) temperature (10, 25, 40, 55, 70 °C), time (0. 60. 120, 180, 240 min), the volume of extract (0.5, 1.5, 2.5, 3.5, 4.5 mL), and volume of AgNO_3_ solution (15, 20, 25, 30, 35 mL). Peak intensity, which corresponds to the surface plasmon resonance of silver nanoparticles was set as the dependent variable. Optimal conditions for the synthesis of silver nanoparticles using *L. bicolor* aqueous extract were attained after obtaining the set criteria.

#### 2.5.2 Verification of Central Composite Design Model

Predicted optimal conditions (pH = 10, temperature = 55^°^C, time = 180 min, volume of extract = 1.5 mL, and volume of AgNO_3_ = 20 mL) from the model were used in an actual experiment. A color change from clear light yellow to a dark brown colloidal solution without aggregations was observed. Using UV-Vis spectroscopy, a high-intensity and narrow SPR peak at 420 nm was observed.

## Ethics Statements

N/A

## CRediT authorship contribution statement

**Krishia Rei A. Javier:** Investigation, Software, Methodology, Writing – original draft. **Drexel H. Camacho:** Resources, Writing – review & editing, Supervision.

## Declaration of Competing Interest

The authors declare that they have no known competing financial interests or personal relationships that could have appeared to influence the work reported in this paper.

The authors declare the following financial interests/personal relationships which may be considered as potential competing interests.

## Data Availability

Dataset on the optimization by response surface methodology for the synthesis of silver nanoparticles using Laxitextum bicolor mushroom (Original data) (Mendeley Data).
